# Perceptions of Intraoperative Music Among Surgical Trainees and Operating Room Staff: A Cross-Sectional Study in an Accreditation Council for Graduate Medical Education (ACGME)-Accredited Program

**DOI:** 10.7759/cureus.101331

**Published:** 2026-01-12

**Authors:** Juan R Medina-Morell, Fabian Ramirez-Rivera, Madelyn Zapata-Valentin, Adriana Bolanos-Rios, Rafael Santini-Dominguez

**Affiliations:** 1 General Surgery, Centro Medico Episcopal San Lucas, Ponce, PRI; 2 Surgery, Ponce Health Sciences University, Ponce, PRI; 3 Epidemiology and Public Health, Centro Medico Episcopal San Lucas, Ponce, PRI; 4 General Surgery, St. Luke's Episcopal Medical Center, Ponce, PRI

**Keywords:** general surgery, intraoperative music, music types, operating room environment, perioperative music, resident performance, surgical training, medical education

## Abstract

Background: Music has been shown to influence cognitive performance and emotional regulation, yet its role within the surgical environment remains incompletely understood. In the operating room (OR), music may enhance mood and teamwork, but concerns persist regarding distraction and communication. This study evaluated perceptions of intraoperative music among surgical trainees and perioperative staff within an academic training environment.

Objective: This study aimed to assess the perceived effects of intraoperative music on mood, focus, communication, and performance among surgical trainees, incorporating perspectives from attending surgeons, nurses, anesthetists, and OR personnel.

Methods: This cross-sectional study was conducted at Centro Médico Episcopal San Lucas between October and November 2024. A total of 107 participants, including attending physicians, surgical residents, nurses, anesthetists, surgical technologists, and administrative OR staff, were surveyed via Research Electronic Data Capture (Vanderbilt University, Nashville, TN). A Likert scale was used to assess perceptions of music’s influence on mood, focus, communication, and resident performance.

Results: All 107 participants reported exposure to music in the OR, and overall perceptions were largely favorable. Among residents, 25 of 29 (86.2%) reported that music improved their mood, and 21 (72.4%) indicated that it helped them feel calmer during procedures. Improved focus was reported by 16 residents (55.2%), while 12 (41.4%) indicated some degree of distraction. Only three residents (10.3%) reported having committed an error they attributed to music, and one (3.4%) reported witnessing such an event. The majority of residents, 21 (72.4%), disagreed that music prolonged operative time.

Faculty responses demonstrated similar trends. Most faculty members reported that music improved residents’ mood (17 of 20, 85%) and enhanced focus during procedures (12 of 20, 60%). Although a minority expressed concern regarding distraction or communication interference, the majority did not perceive music as detrimental. Only five faculty (25%) reported ever requesting that music be turned off, and none reported music-related operative errors.

Conclusions: This study shows that intraoperative music is generally perceived by trainees and faculty as beneficial, particularly for enhancing mood and promoting calmness. While many residents reported improved focus, a notable subset experienced overstimulation and communication challenges, especially during complex procedures. These findings highlight the need for context-sensitive use of music, guided by procedural demands and team consensus. To validate and optimize its role in surgical education, future studies should incorporate objective performance metrics and explore standardized approaches to the use of music.

## Introduction

Music has long played a role in modulating human mood and optimizing performance under pressure. In sports psychology, music has been demonstrated to elevate motivation, enhance physical endurance, and improve fine motor coordination [1,2]. It has also been linked to reduced perceived exertion and improved concentration during repetitive or high-stakes tasks. These ergogenic effects raise the question of whether similar benefits can be harnessed in the operating room (OR), a domain that demands intense focus, procedural fluency, and emotional resilience. The OR carries life-or-death implications, making it critical to understand not only potential benefits but also risks of distraction associated with music, including overstimulation or delayed response to alarms [3].

The OR environment is typically saturated with auditory stimuli, including alarms, conversations, and the sounds of surgical equipment. Studies have shown that ambient noise in hospitals frequently exceeds World Health Organization guidelines, which recommend maximum noise levels of 35 dB in hospital settings [3]. In practice, noise in the OR typically ranges from 55 to 65 dB and may exceed 80 dB, particularly during orthopedic and trauma procedures [4]. These noise levels are comparable to heavy traffic and have been linked to increased stress, impaired communication, and reduced task performance among surgical staff. High background noise can also compromise patient safety by masking alarms or delaying team responses to intraoperative changes [5]. Thus, strategies to mitigate auditory overload while maintaining focus are increasingly relevant in surgical practice.

Conversely, music has been proposed as a cognitive buffer against stress, potentially improving attention, communication, and collaboration within the OR team. Research has identified positive effects of music on postoperative recovery, including reductions in pain perception, heart rate, anxiety, and opioid use [5,6]. Surgeons who listen to preferred music during operations have reported enhanced mood, reduced perceived effort, and even lower stress biomarkers [7]. Notably, randomized trials have shown that senior surgical residents demonstrate improved performance in simulated closures when listening to music, while junior residents may be more susceptible to distraction and error [8,9]. These findings suggest that the impact of music is not uniform but may depend on experience level, case complexity, and contextual variables such as volume, tempo, and genre.

Despite these promising observations, the literature remains limited regarding how intraoperative music impacts the learning experience of surgical trainees. Most existing studies focus on surgeon performance or patient outcomes, leaving a gap in understanding the psychological and cognitive implications for residents in training, who are simultaneously acquiring technical skills and managing stress in a high-pressure environment. Furthermore, few studies integrate the perspectives of multiple OR personnel, such as nurses, anesthetists, and technologists, who not only interact closely with trainees but also help maintain effective team dynamics. The perspectives of these groups are critical, as their observations provide insight into whether music fosters collaboration or inadvertently hampers communication.

This study seeks to address these gaps by examining the perceived effects of intraoperative music on surgical trainees within an Accreditation Council for Graduate Medical Education (ACGME)-accredited program. We assess not only resident self-reports but also faculty and staff perceptions, aiming to provide a multifaceted view of music’s influence on residents’ focus, mood, and performance. By including voices from across the OR team, this study offers a broader understanding of how music functions in the training environment. Moreover, as a pilot project, it establishes the foundation for future research phases involving direct performance measurement and physiological stress markers, with the ultimate goal of informing evidence-based guidelines on music use in surgical education and practice.

## Materials and methods

Study design and setting

A cross-sectional prospective study was conducted at Centro Médico Episcopal San Lucas in Puerto Rico from October to November 2024, encompassing a broad range of surgical specialties and procedural disciplines. The hospital operates under ACGME accreditation and houses a multidisciplinary OR workforce, including General Surgery Residents, Urology Residents, Cardiology Fellows, Attending Physicians, Nursing Staff, Anesthetists, OR Technicians, and Administrative Personnel. This diverse OR team allowed for the collection of perceptions across multiple roles, capturing both trainee and staff perspectives on intraoperative music. The study aimed to evaluate the perceived effects of music on mood, concentration, communication, and procedural performance, providing a comprehensive understanding of its role within the surgical training environment.

Participant recruitment and survey instrument

Email invitations were sent to 209 staff members for recruitment. Participants could access the survey via a QR code generated by a secure web application, which was available on flyers posted in common OR areas and through the emails sent. Eligible participants included surgical trainees (general surgery, cardiology, and urology), attending physicians, OR nurses, anesthetists, OR technicians, and administrative personnel. Inclusion criteria consisted of individuals actively involved in surgical procedures relating participation of residents within the institution’s ACGME-accredited programs. Exclusion criteria included participants with no direct contact with residents or those who declined to participate in the study.

The survey, created and distributed, consisted of 28 questions (see the Appendix). These addressed demographic variables (age, gender, role, specialty), general music preferences, and intraoperative listening habits. Likert-scale and binary response formats were used to assess the perceived impact of music on mood, concentration, communication, and performance. The questionnaire included branching logic to ensure appropriate questions were shown based on participant role (e.g., residents assessed themselves; faculty and staff evaluated trainees). The instrument underwent internal validation through pilot testing with 10 OR staff prior to full deployment. The complete survey is available as a supplementary file.

Ethical considerations and data analysis

This study was initially conducted as an internal quality improvement project at the hospital in October-November 2024 and received Institutional Review Board (IRB) approval in April 2025, prior to submission for publication. The study was reviewed and approved by the IRB of Ponce Health Sciences University (protocol no.: 2503248278) and was deemed exempt from full review. All procedures were conducted in accordance with institutional ethical standards and the Declaration of Helsinki. Participation was voluntary, and no identifiable data were collected. Responses were coded and analyzed using IBM Statistical Package for the Social Sciences Statistics version 28 (IBM Corp., Armonk, NY). Descriptive statistics characterized the sample, while chi-square and independent samples t-tests assessed differences across roles and demographics. Statistical significance was set at p < 0.05 with 95% confidence intervals.

## Results

Demographics and participant roles

The final sample included 107 respondents (51.2% response rate). Gender distribution was nearly equal, with 53 (49.5%) male and 54 (50.5%) female participants. Most respondents were between 30 and 39 years (43, 40.2%), followed by 20-29 years (26, 24.3%), 40-49 years (18, 16.8%), 50-59 years (12, 11.2%), and 60 years or older (8, 7.5%). One hundred seven respondents reported role in the OR: residents (29, 27.2%), faculty (20, 18.6%), nurses (23, 21.4%), anesthetists (14, 13.1%), OR technicians (16, 14.9%), and administrative staff (5, 4.8%). Regarding specialty affiliation, most respondents were associated with general surgery (76, 71%), followed by obstetrics-gynecology (12, 11.2%), cardiology (14, 13.1%), and urology (5, 4.7%) (Table 1).

**Table 1:** Participant demographics (n = 107) OR: operating room; OB-GYN: obstetrics and gynecology

Variable	n (%)
Gender	
Male	53 (49.5%)
Female	54 (50.5%)
Age group	
20-29 years	26 (24.3%)
30-39 years	43 (40.2%)
40-49 years	18 (16.8%)
50-59 years	12 (11.2%)
60+	8 (7.5%)
Role in OR	
Resident	29 (27.2%)
Faculty	20 (18.6%)
Nurse	23 (21.4%)
Anesthetist	14 (13.1%)
OR technician	16 (14.9%)
Administrative staff	5 (4.8%)
Specialty	
General surgery	76 (71%)
OB-GYN	12 (11.2%)
Cardiology	14 (13.1%)
Urology	5 (4.7%)

Music exposure and preferences

Participants reported diverse musical preferences and patterns of intraoperative listening that varied by role (Table 2). Residents most frequently preferred Salsa, Reggaeton, and Pop, listened to music in the OR for an average of two to three hours per day, and most often held decision-making authority over music selection (53, 49.5%). Faculty tended to favor Classical, Jazz, and Rock, listened one to two hours per day, and reported control over music selection in 41 (38.3%). Nurses commonly preferred Pop, Reggaeton, and Salsa, listened to two to three hours per day, but only six (5.6%) indicated having authority over music choice. Anesthetists preferred Electronic, Jazz, and Rock, reported listening durations of three to four hours per day, and five (4.7%) had decision-making control. OR technicians most often selected Reggaeton, Hip-Hop, and Salsa, listened to two to three hours per day, with two (1.9%) reporting influence over music selection. Administrative staff demonstrated varied musical preferences, averaged one hour of intraoperative listening per day, and 0 (0%) reported decision-making authority. Overall, residents and faculty represented the majority of those with control over intraoperative music selection, whereas other staff roles experienced similar listening exposure but had limited influence.

**Table 2:** Music preferences by professional role (genres, listening habits, and decision-making authority) OR: operating room

Role in OR	Top genres preferred	Average listening time in OR	Authority on music selection frequency, n (%)
Residents	Salsa, Reggaeton, Pop	2-3 hours/day	53 (49.5%)
Faculty	Classical, Jazz, Rock	1-2 hours/day	41 (38.3%)
Nurses	Pop, Reggaeton, Salsa	2-3 hours/day	6 (5.6%)
Anesthetist	Electronic, Jazz, Rock	3-4 hours/day	5 (4.7%)
OR technicians	Reggaeton, Hip-Hop, Salsa	2-3 hours/day	2 (1.9%)
Admin staff	Various	1 hour/day	0 (0%)
Total responses	-	-	107 (100%)

Resident's psychological and cognitive effects

Participants generally reported positive psychological effects of music in the OR. Most respondents indicated that music improved their mood, with 16 (55.2%) strongly agreeing, nine (31%) agreeing, only four (13.8%) were neutral, and none disagreed. Similarly, 10 (34.5%) strongly agreed, and 11 (37.9%) agreed that music helped them feel calmer during procedures. Regarding concentration, nine (31%) agreed, and seven (24.1%) strongly agreed that music enhanced focus, while eight (27.6%) were neutral, and five (17.2%) disagreed or strongly disagreed. When asked whether music disrupted concentration, most participants disagreed or strongly disagreed, 12 (41.4%), whereas 11 (37.9%) were neutral. Perceived effects on surgical performance were generally positive: nine (31%) strongly agreed, and eight (27.6%) agreed that music enhanced performance, while 10 (34.5%) were neutral. Regarding case complexity, nearly half of the respondents (14, 48.3%) were neutral on whether music should be avoided during complex procedures, while eight (27.6%) disagreed or strongly disagreed. Finally, most participants did not believe music prolonged operative time, with 21 (72.4%) disagreeing or strongly disagreeing. Overall, music was largely perceived as beneficial for mood, calmness, focus, and performance, although a minority reported distraction or overstimulation, particularly in high-complexity cases (Table 3).

**Table 3:** Resident perceptions of music’s impact on focus, mood, and performance

Perception statement	Strongly disagree, n (%)	Disagree, n (%)	Neutral, n (%)	Agree, n (%)	Strongly agree, n (%)	Total, n (%)
Music improves my mood during surgery	0 (0%)	0 (0%)	4 (13.8%)	9 (31%)	16 (55.2%)	29 (100%)
Music helps me feel calmer during procedures	1 (3.4%)	1 (3.4%)	6 (20.7%)	11 (37.9%)	10 (34.5%)	29 (100%)
Music enhances my focus while operating	1 (3.4%)	4 (13.8%)	8 (27.6%)	9 (31%)	7 (24.1%)	29 (100%)
Music disrupts my concentration	6 (20.7%)	6 (20.7%)	11 (37.9%)	4 (13.8%)	2 (6.9%)	29 (100%)
Music enhances my surgical performance	1 (3.4%)	1 (3.4%)	10 (34.5%)	8 (27.6%)	9 (31%)	29 (100%)
Music should not be played during complex cases	2 (6.9%)	6 (20.7%)	14 (48.3%)	3 (10.3%)	4 (13.8%)	29 (100%)
Music prolongs procedure time	10 (34.5%)	11 (37.9%)	5 (17.2%)	2 (6.9%)	1 (3.4%)	29 (100%)

Music-related overstimulation and errors

When examining overstimulation and error-related perceptions among residents and faculty, 18 residents (62.1%) reported having requested that music be turned off due to overstimulation, compared with five faculty members (25%), a difference that was statistically significant (p = 0.019). Residents also more frequently reported witnessing an attending physician turn off the music, with 15 residents (51.7%) compared with 10 faculty members (50%), although this difference was not statistically significant (p = 0.21). Perceptions of communication disruption were low overall, with five residents (17.2%) and three faculty (15%) indicating interference (p = 0.78). Music-related errors were uncommon: one resident (3.4%) reported witnessing an intraoperative error attributed to music, and three residents (10.3%) reported personally committing such an error, while no faculty reported any music-related errors (p = 0.071-0.091). Overall, these findings suggest that although most participants tolerated intraoperative music well, residents experienced greater perceived overstimulation and were more likely than faculty to request temporary cessation of music (Table 4).

**Table 4:** Comparison of music-related overstimulation and error reports by resident and faculty self-reports Percentages are based on the total responses ("Yes") answered by premise, not on the sum of all responses OR: operating room

Incident	Residents, n (%)	Faculty, n (%)	p value
Requested music to be turned off due to overstimulation	18 (62.1%)	5 (25%)	0.019
Witnessed the attending physician turn off the music	15 (51.7%)	10 (50%)	0.21
Believe music disrupts communication in the OR	5 (17.2%)	3 (15%)	0.78
Witnessed an intraoperative error due to music	1 (3.4%)	0 (0%)	0.071
Personally committed an error related to music	3 (10.3%)	0 (0%)	0.091
Total per-group response	29 (100%)	20 (100%)	-

Perceived effects of music on surgical performance and communication

Participants across different OR roles generally reported that music did not substantially prolong surgical procedures or interfere with communication, even during complex cases (see Table 5). Only three residents (10.3%), one faculty member (5%), and two nurses (8.7%) felt that music made procedures take longer (p = 0.62). Among complex cases, seven residents (24.1%), five faculty (25%), and five nurses (21.7%) believed that music should not be played, whereas the majority did not share this concern (p = 0.89). Music was reported to help maintain focus during longer procedures by 19 residents (65.5%), 11 faculty (55%), and 14 nurses (60.9%) (p = 0.74). Disruption of communication in complex cases was infrequently reported, with five residents (17.2%), three faculty (15%), and four nurses (17.4%) indicating interference (p = 0.83). Overall, these results suggest that music is generally perceived as supportive of concentration and workflow without major negative effects on procedure duration or team communication.

**Table 5:** Perceptions of music on procedure length, focus, and communication by OR role Percentages are based on the total responses ("Yes") answered by premise, not on the sum of all responses OR: operating room

Perception statement	Residents, n (%)	Faculty, n (%)	Nurses, n (%)	p value
Music makes procedures take longer	3 (10.3%)	1 (5%)	2 (8.7%)	0.62
Music should not be played during complex cases	7 (24.1%)	5 (25%)	5 (21.7%)	0.89
Music helps maintain focus in long procedures	19 (65.5%)	11 (55%)	14 (60.9%)	0.74
Music disrupts communication in complex cases	5 (17.2%)	3 (15%)	4 (17.4%)	0.83
Total per-group response	29 (100%)	20 (100%)	23 (100%)	-

Faculty responses regarding the impact of music on residents varied across several domains (see Table 6). Music was perceived to have a positive effect on resident mood by 12 faculty (60%) who agreed and five (25%) who strongly agreed, while two (10%) were neutral and one (5%) disagreed. Similarly, nine faculty (45%) agreed and five (25%) strongly agreed that music helped residents feel calmer during procedures; five (25%) were neutral, and one (5%) disagreed. Regarding concentration, eight faculty (40%) agreed and four (20%) strongly agreed that music improved residents’ focus, while six (30%) were neutral and two (10%) disagreed. Perceived enhancement of surgical performance followed a similar pattern, with eight faculty (40%) agreeing and seven (35%) strongly agreeing, while four (20%) were neutral and one (5%) disagreed. Concerns about communication interference were less common: eight faculty (40%) disagreed and five (25%) strongly disagreed that music disrupted communication in the OR, while four (20%) were neutral and only three (15%) agreed or strongly agreed. Likewise, seven faculty (35%) disagreed and six (30%) strongly disagreed that music should not be played during complex cases, with smaller proportions agreeing (2, 10%) or strongly agreeing (1, 5%). Similar distributions were observed regarding whether music prolonged procedural time, with seven (35%) disagreeing and six (30%) strongly disagreeing, and only two (10%) agreeing. Finally, when asked whether they had ever instructed residents to turn music off during a case, responses were evenly split: 10 faculty (50%) reported doing so, while 10 (50%) had not.

**Table 6:** Faculty evaluations of music’s impact on resident mood, calmness, concentration, and surgical performance OR: operating room

Perception statement	Strongly disagree, n (%)	Disagree, n (%)	Neutral, n (%)	Agree, n (%)	Strongly agree, n (%)	Total, n (%)
Music positively influences residents' mood	0 (0%)	1 (5%)	2 (10%)	12 (60%)	5 (25%)	20 (100%)
Music helps residents feel calmer during procedures	0 (0%)	1 (5%)	5 (25%)	9 (45%)	5 (25%)	20 (100%)
Music improves residents' concentration	0 (0%)	2 (10%)	6 (30%)	8 (40%)	4 (20%)	20 (100%)
Music enhances residents' surgical performance	0 (0%)	1 (5%)	4 (20%)	8 (40%)	7 (35%)	20 (100%)
Music disrupts communication in the OR	5 (25%)	8 (40%)	4 (20%)	1 (5%)	2 (10%)	20 (100%)
Music should not be played during complex cases	6 (30%)	7 (35%)	4 (20%)	2 (10%)	1 (5%)	20 (100%)
Music prolongs procedure time	6 (30%)	7 (35%)	4 (20%)	2 (10%)	1 (5%)	20 (100%)
I have asked residents to turn off the music during a case	0 (0%)	10 (50%)	0 (0%)	10 (50%)	0 (0%)	20 (100%)

Gender-based perceptions

Analysis of gender differences revealed distinct patterns in how male and female participants perceived intraoperative music (see Table 7). Music was reported to help maintain calmness during procedures by 38 male participants (71.7%) compared with 25 female participants (46.3%) (p = 0.049). Similarly, 27 male participants (50.9%) indicated that music enhanced their focus while operating, compared with 20 female participants (37%) (p = 0.005). Music was also perceived to improve surgical performance by 24 male participants (45.3%), whereas this was reported by 15 female participants (27.8%) (p = 0.004). In contrast, disruption of concentration was more frequently reported among female participants, with 12 (22.2%) indicating interference compared with seven male participants (13.2%) (p = 0.21). A similar pattern was seen regarding complex cases, in which 15 female participants (27.8%) and 11 male participants (20.8%) believed music should not be played (p = 0.37). Finally, 32 female participants (59.3%) and 25 male participants (47.2%) reported having requested music be turned off due to overstimulation, although this difference was not statistically significant (p = 0.18).

**Table 7:** Gender-based differences in perceptions of intraoperative music Percentages are based on the total responses ("Yes") answered by premise, not on the sum of all responses

Perception statement	Male, n (%)	Female, n (%)	p value
Music helps me feel calmer during procedures	38 (71.7%)	25 (46.3%)	0.049
Music enhances my focus while operating	27 (50.9%)	20 (37%)	0.005
Music improves my surgical performance	24 (45.3%)	15 (27.8%)	0.004
Music disrupts my concentration	7 (13.2%)	12 (22.2%)	0.21
Music should not be played during complex cases	11 (20.8%)	15 (27.8%)	0.37
I have requested that the music be turned off due to overstimulation	25 (47.2%)	32 (59.3%)	0.18
Total per-group response	53 (100%)	54 (100%)	-

Differences in perspectives among faculty by age group

Analysis of faculty perceptions across age groups revealed notable trends regarding the impact of music on resident training (see Table 8). All faculty aged ≥60 years (5, 100%) agreed that music improved residents’ mood and helped them feel calmer during procedures, compared with five faculty aged 40-59 years (71.4%) and five faculty <40 years (62.5%) for mood, and four faculty aged 40-59 years (57.1%) and four faculty <40 years (50%) for calmness; both differences were statistically significant (p = 0.02). Older faculty also more frequently perceived music as beneficial for resident focus and surgical performance, with four faculty ≥60 years (80%) endorsing improved focus and performance, compared with four faculty aged 40-59 years (57.1%) and five faculty (71.4%), and three faculty <40 years (37.5%) and four faculty (50%), respectively; however, these differences were not statistically significant (p = 0.07 and p = 0.12). Perceptions of communication disruption were low across all age groups, reported by two faculty <40 years (25%), one aged 40-59 years (14.3%), and zero ≥60 years (0%). Similarly, beliefs that music should not be played during complex cases were uncommon, reported by three faculty <40 years (37.5%), two aged 40-59 years (28.6%), and one ≥60 years (20%) (p = 0.32). Requests to turn off music were reported at similar rates across age groups: four faculty <40 years (50%), three aged 40-59 years (42.9%), and two ≥60 years (40%) (p = 0.89), indicating that age did not meaningfully influence direct intervention during surgery.

**Table 8:** Faculty perspectives on music in the OR by age group Percentages are based on the total responses ("Yes") answered by premise, not on the sum of all responses OR: operating room

Perception statement	<40 years, n (%)	40-59 years, n (%)	>60 years, n (%)	p value
Music improves residents' mood	5 (62.5%)	5 (71.4%)	5 (100%)	0.02
Music helps residents feel calmer	4 (50%)	4 (57.1%)	5 (100%)	0.02
Music enhances residents' focus	3 (37.5%)	4 (57.1%)	4 (80%)	0.07
Music improves residents' surgical performance	4 (50%)	5 (71.4%)	4 (80%)	0.12
Music disrupts communication in the OR	2 (25%)	1 (14.3%)	0 (0%)	0.18
Music should not be played during complex cases	3 (37.5%)	2 (28.6%)	1 (20%)	0.32
I have asked residents to turn off the music	4 (50%)	3 (42.9%)	2 (40%)	0.89
Total per-group response	8 (100%)	7 (100%)	5 (100%)	-

## Discussion

This study explored the perceptions of intraoperative music among surgical trainees and OR staff in an ACGME-accredited program, incorporating perspectives across roles, gender, and faculty age. Overall, findings indicate that music is widely perceived as beneficial, particularly for enhancing mood, calmness, and concentration, although responses varied among residents, faculty, nurses, anesthetists, and technicians. Importantly, while most respondents viewed music positively, a subset reported overstimulation or distraction, suggesting the need for context-sensitive application in the OR.

Our results confirm and extend prior research demonstrating the psychological and performance-related benefits of music in surgery. As shown in Table 3, music was widely perceived to improve intraoperative mood: 86 respondents (80.4%) reported that it enhanced their emotional state, and 64 respondents (59.8%) reported that it improved their focus. These findings mirror studies reporting reductions in anxiety, improved concentration, and improved technical task performance in music-assisted conditions [8,10]. While responses were generally positive across roles, residents demonstrated particularly strong endorsement of the benefits of music on focus (62.1%), suggesting its influence may be more pronounced during early training or repetitive procedural tasks.

Faculty responses further supported these advantages. In Table 6, 12 faculty (60%) agreed, and five (25%) strongly agreed that music positively influenced residents’ mood, and eight faculty (40%) agreed, and seven (35%) strongly agreed that music enhanced resident surgical performance. These perceptions align with experimental studies demonstrating reductions in physiologic stress and improved procedural fluency under musical conditions [11-14]. Interestingly, faculty tended to view the impact of music on residents more favorably than residents themselves, underscoring the potential influence of experience, familiarity with the OR environment, and differing expectations across training levels.

Meaningful differences emerged across OR roles. As reported in Table 2, residents and nurses reported the longest daily exposure to intraoperative music (two to three hours/day), and residents held the greatest decision-making authority over music selection, with 53 residents (49.5%) indicating control, substantially more than nurses (6%) or technicians (1.9%). Faculty who preferred more traditional genres such as Classical, Jazz, or Rock reported less frequent control over music selection (41 faculty, 38.3%), consistent with findings that music authority in the OR is often trainee-driven and generationally stratified [4,15]. These dynamics highlight how hierarchy and generational preferences may shape auditory environments in training hospitals.

Despite overall positive perceptions, concerns related to overstimulation and distraction were evident, particularly among residents. Eighteen residents (78.3%) reported having asked for music to be turned off due to overstimulation, compared with only five faculty members (21.7%), a statistically significant difference. Residents also more frequently reported witnessing an attending physician turn off the music (15 residents vs. 10 faculty), although this difference was not significant. Although music-related errors were rare, one resident (3.4%) reported witnessing a music-related error, and three residents (10.3%) reported personally committing one, while no faculty indicated any such events. These findings are consistent with existing literature showing that, although music can improve technical efficiency, it may interfere with auditory attentiveness and communication clarity under specific conditions [3,16]. Notably, faculty tended to underestimate resident-reported overstimulation, suggesting that trainees may experience a higher cognitive load that is not always apparent to supervising surgeons [11,17]. This highlights that while music is broadly well tolerated, its effects are not uniform and may be influenced by experience level, task complexity, and individual sensory tolerance.

Gender and faculty age also influenced perceptions. As shown in Table 7, male respondents more frequently endorsed benefits on mood, focus, and performance (e.g., 38 male respondents (71.1%) vs. 25 female respondents (46.3%) reporting improved calmness; p = 0.049), whereas female respondents more often requested music cessation due to overstimulation (32 female respondents (59.3%) vs. 25 male respondents (47.2%); p = 0.18). This variability reflects potential differences in sensory tolerance, consistent with broader literature on music perception and individual thresholds for auditory stimulation [1,2]. In Table 8, older faculty (≥60 years) expressed uniformly positive beliefs in music’s impact, with five of five (100%) endorsing improvements in mood and calmness, compared with five of seven (71.4%) faculty aged 40-59 and five of eight (62.5%) under age 40 (p = 0.02), suggesting the influence of experience, generational norms, and workload patterns in shaping perceptions [3,4,12].

Taken together, the findings indicate that music in the OR is neither uniformly beneficial nor uniformly disruptive but instead functions as a context-dependent tool shaped by team dynamics, workflow intensity, individual sensory tolerance, and role hierarchy. Practical recommendations supported by our data and prior literature include maintaining music at a low to moderate volume, pausing it during high-stakes phases, and involving all OR members in playlist curation to foster inclusivity and psychological safety [11,16-18]. By recognizing these nuances, intraoperative music can be managed thoughtfully, optimizing its benefits while minimizing potential risks.

Practical implications

Music use in the OR should be selective, context-sensitive, and tailored to the needs of both the procedure and the surgical team. Evidence indicates that maintaining music at a low to moderate volume helps reduce distractions and cognitive interference, thereby supporting surgical precision and communication [11,17]. Preoperative discussions regarding music preferences and acceptable volume are recommended to establish mutual agreement among team members and avoid conflict or dissatisfaction [16]. During surgery, volume should remain especially low during high-stakes or technically demanding steps to ensure that critical instructions and alarms are clearly heard [3,12]. The lead surgeon should also have the discretion to pause or stop music altogether when performing complex or unfamiliar tasks, prioritizing patient safety above preference [9]. Importantly, playlist selection should not be unilateral; involving residents, nurses, and anesthetists in curating music fosters inclusivity, teamwork, and psychological safety, while also enhancing morale and reducing stress in the OR environment [1,16,18]. Taken together, these strategies highlight that music can be beneficial in the OR when managed thoughtfully, but it requires clear guidelines, respect for team dynamics, and patient-centered priorities.

Strengths and limitations

This study’s strength lies in its broad sampling across OR roles, offering a 360° view of the training environment. Capturing perspectives from residents, faculty, anesthetists, nurses, and technicians provides a unique lens on team dynamics rarely described in the literature. However, reliance on subjective perceptions limits the generalizability of findings, and the 51.2% response rate introduces the possibility of selection bias. Additionally, performance evaluations by nonsurgeons (e.g., Certified Registered Nurse Anesthetist, technicians, and administrative staff) may lack objectivity, reflecting perceptions rather than validated measures of surgical skill. While these diverse viewpoints enrich understanding, they also introduce interpretive variability.

Future directions

Future research should incorporate qualitative interviews to better understand specific scenarios in which music was perceived to enhance or hinder performance [11]. Objective assessments through controlled trials, such as measuring task completion time, accuracy, and error rates during simulated procedures, would help quantify music’s impact on surgical performance [12,18]. Additionally, studies examining the effects of decibel level, tempo, and musical genre could help define optimal parameters for intraoperative music [17,18]. Prior work evaluating stress using physiological markers such as heart rate variability and blood pressure under musical versus nonmusical conditions provides a useful model for integrating subjective and objective measures [11,12]. Expanding this work to multi-institutional and cross-cultural contexts would further strengthen generalizability and inform the development of standardized guidelines.

## Conclusions

Intraoperative music is generally perceived by trainees and faculty as beneficial, particularly for enhancing mood, calmness, and focus. Many residents reported improved concentration and a more positive emotional state during procedures, although a subset experienced overstimulation or minor communication challenges, especially during complex or high-stakes cases. These findings highlight that music in the OR is context-dependent, requiring thoughtful implementation tailored to the procedure, team dynamics, and individual tolerance.

When applied judiciously, music can support psychological well-being, promote team cohesion, and enhance the overall training environment. Preoperative discussion of preferences, shared control over playlists, and careful volume management during critical surgical steps are practical strategies to maximize benefits while minimizing distraction. Importantly, these findings suggest that music may also serve as a subtle tool for stress reduction and resilience-building in surgical trainees, potentially improving long-term performance and retention of skills. Incorporating structured approaches to music use could further enhance OR culture by fostering inclusivity and mutual respect among team members. Overall, intraoperative music represents a low-cost, accessible tool that, when managed thoughtfully, can enhance surgical training, team dynamics, and well-being in the OR.

## Appendices

**Table 9:** Survey provided to participants OR: operating room; OB-GYN: obstetrics and gynecology

Section	Item	Response options
Demographics	Age	Less than 30, 30-40, 41-50, 51-60, More than 60
	Gender	Male, Female, Non-Binary, Prefer not to say
	Role	Faculty, Resident, Nurse, Anesthetist, Operating Room Tech, Administrative role
	Resident year	PGY1, PGY2, PGY3, PGY4, PGY5, PGY6
	Specialty	Surgery, OB-GYN, Urology, Cardiology
Preferences	Do you like to listen to music?	Yes, No
	How many hours a day do you spend listening to music?	None, <1 hour, 1-3 hours, 4-5 hours, >5 hours
	Of those hours, how many are spent listening to music in the OR?	None, <1 hour, 1-3 hours, 4-5 hours, >5 hours
	In which situations do you listen to music?	Commuting, Working/Studying, Exercising, Relaxing, Social Gathering
	What genres of music do you listen to during surgical procedures?	None, Pop, Rock, Hip-Hop, Classical, Jazz, Electronic, Salsa, Reggaeton, Reggae
Resident self-evaluation	Music improves my mood in the OR	Strongly Agree, Agree, Neutral, Disagree, Strongly Disagree
	Music helps improve my concentration during surgery	Strongly Agree, Agree, Neutral, Disagree, Strongly Disagree
	Music makes me feel calmer during surgery	Strongly Agree, Agree, Neutral, Disagree, Strongly Disagree
	Music impacts my performance positively during surgery	Strongly Agree, Agree, Neutral, Disagree, Strongly Disagree
	Music disrupts communication in the OR	Strongly Agree, Agree, Neutral, Disagree, Strongly Disagree
	Music should not be played during complex procedures	Strongly Agree, Agree, Neutral, Disagree, Strongly Disagree
	Music makes me take longer during surgical procedures	Strongly Agree, Agree, Neutral, Disagree, Strongly Disagree
	Has an attending ever asked you to turn off the music during a surgical procedure?	Strongly Agree, Agree, Neutral, Disagree, Strongly Disagree
Faculty evaluation	Music improves resident's concentration during surgery	Strongly Agree, Agree, Neutral, Disagree, Strongly Disagree
	Music affects resident's mood positively during surgery	Strongly Agree, Agree, Neutral, Disagree, Strongly Disagree
	Music makes residents feel calmer during surgical procedures	Strongly Agree, Agree, Neutral, Disagree, Strongly Disagree
	Music positively impacts resident performance during surgery	Strongly Agree, Agree, Neutral, Disagree, Strongly Disagree
	Music disrupts communication with residents during surgery	Strongly Agree, Agree, Neutral, Disagree, Strongly Disagree
	Music should NOT be played during complicated procedures	Strongly Agree, Agree, Neutral, Disagree, Strongly Disagree
	Music makes residents take longer during procedures	Strongly Agree, Agree, Neutral, Disagree, Strongly Disagree
Additional questions	Have you ever felt the need to turn off the music due to resident overstimulation?	Yes, No
	Have you ever asked peers to turn off music during a procedure?	Yes, No
	Has music caused you to make an error, or have you witnessed an error during surgery due to music?	Yes, No
